# Parenting approaches, family functionality, and internet addiction among Hong Kong adolescents

**DOI:** 10.1186/s12887-016-0666-y

**Published:** 2016-08-18

**Authors:** Cynthia Sau Ting Wu, Ho Ting Wong, Kin Fai Yu, Ka Wing Fok, Sheung Man Yeung, Cheuk Ho Lam, Ka Man Liu

**Affiliations:** 1 GH506, School of Nursing, The Hong Kong Polytechnic University, Hung Hom, Kowloon Hong Kong; 2 Institute of Geographic Sciences and Natural Resources Research, Chinese Academy of Sciences, Beijing, China

**Keywords:** Parenting approaches, Family functionality, Internet addiction, Adolescents, Chinese

## Abstract

**Background:**

Internet addiction (IA) among adolescents has become a global health problem, and public awareness of it is increasing. Many IA risk factors relate to parents and the family environment. This study examined the relationship between IA and parenting approaches and family functionality.

**Methods:**

A cross-sectional study was conducted with 2021 secondary students to identify the prevalence of IA and to explore the association between adolescent IA and familial variables, including parents’ marital status, family income, family conflict, family functionality, and parenting approaches.

**Results:**

The results revealed that 25.3 % of the adolescent respondents exhibited IA, and logistic regression positively predicted the IA of adolescents from divorced families, low-income families, families in which family conflict existed, and severely dysfunctional families. Interestingly, adolescents with restricted Internet use were almost 1.9 times more likely to have IA than those whose use was not restricted.

**Conclusions:**

Internet addiction is common among Chinese adolescents in Hong Kong, and family-based prevention strategies should be aligned with the risk factors of IA.

## Background

In the era of information technology, the Internet has been the most influential agent, having had a huge impact on daily human life. Due to the diverse nature of the Internet and the advantages it offers, its use is becoming far more widespread. In Hong Kong, the percentage of homes with computers connected to the Internet increased from 70.1 % in 2007 to 78.6 % in 2012 [1]. Researchers indicate that Internet use is mainly a home-based activity, and that it has become a part of daily adolescent life in the contemporary world [2, 3]. However, for those who are over-dependent on the Internet and even over-use it, problems may occur when they are no longer capable of controlling their own online activity. Scholars regard this abnormal and pathological behavior as Internet addiction (IA).

Adolescents are more vulnerable than other age groups to using the Internet excessively, given that they are not physically or mentally fully developed [4]. Research indicates that the Internet affects adolescents in four areas: physical health, psychosocial development, academic performance, and family relationships [5, 6]. From a global viewpoint, according to studies conducted in Italy, China, South Korea, and Taiwan, the prevalence of IA among adolescents ranges from 10.7–36.7 % [7–10]. These statistics should alert us to the fact that IA has become an international health issue that cannot be overlooked. In local studies, the prevalence of IA ranges from 6.7–19.1 % [11, 12]; clearly, the situation in Hong Kong is not as grave as elsewhere in the world, but it is undoubtedly a significant concern when considering the health of our adolescents.

Researchers from different fields, including psychology, social sciences, health care, and education, are studying the associated factors that influence the prevalence of IA. Among these studies, one identified factor related to Internet usage that influences adolescent behavior is parenting. According to Miller and Plant [13], the degree of parenting relates strongly to adolescent behavior. It is evident that parental guidance reduces the influence of alcohol advertising on adolescents, thus reducing their consumption [14]. Other studies prove that parents act as positive role models and protect their adolescent children from IA [7, 15].

### Internet addiction (IA)

Traditionally, the term *addiction* was developed from a medical model, and it is defined as a psychological and physical dependency on a specific matter [16]. However, until now, no conclusive term has been adopted as the operational definition of IA. Such a term should also include reference to a broadened range of behavioral patterns [17–20].

According to Young [21], IA comprises compulsive behaviors related to any online activities that disturb normal daily life and induce stress on social relationships. With the increasing popularity of the Internet, it is easy for adolescents to have an “uncontrollable urge” to use it [22]. Young further explains that these kinds of urges make adolescents obsessed with the Internet, leading them to use it day and night, regardless of the occurrence of side effects. In 1998, Young developed an assessment tool for IA by adapting Diagnostic and Statistical Manual-IV (DSM-IV) criteria for pathological gambling; this tool is still widely adopted in investigating IA [23]. Moreover, recent research has studied different factors of IA and revealed that age, gender, education level, family support, family conflict, and peer group influence are related to IA [11, 24, 25]. Although Young’s assessment tool for IA is widely used by different researchers, it would be ideal to update the assessment tool when internet addiction disorder (IAD) is included in the newer version of DSM. However, this is not likely to happen in the near future, as only the most specific internet gaming disorder was added to the latest version of DSM-V in 2013, not the more general term of internet addition disorder. The reason for only adding internet gaming disorder was because research has shown that gaming disorder appears to be distinct from other online activities. Moreover, research on other online activities and their adverse effects were also less well documented [26].

### Parents’ role

Family has an important role in influencing the socialization of adolescents [27]. It has been shown that the family is a protective factor in preventing adolescents from taking part in problematic and hazardous behavior, for example, the use of tobacco, illicit substances, and alcohol, and unsafe sexual practices [13, 28]. It has been reported that similar family factors are associated with both substance use and IA, which indicates that these can be grouped under the term of behavioral problems syndrome [29]. Furthermore, adverse conditions like a broken family, family conflict, and low family functionality are reported to be associated with adolescent IA [30].

Several types of parenting approaches are discussed in the literature. Parental monitoring and parental style are the more common parental skills in the daily life of adolescents, rather than paying specific attention to particular types of adolescent activities. Parental monitoring means supervision of the daily life of an adolescent [31], parental style focuses on the overall parenting climate at home [16], while parental guidance is more specific to one kind of activity. Parental guidance refers to setting rules, giving direction, counseling, advising, making a clear distinction between right and wrong, and providing protection in daily activities [32, 33]. However, how these factors relate to IA has not yet been studied extensively, or, in other words, has not been Internet-specific.

The concept of parental guidance refers to a variety of behaviors employed by parents to support their child’s performance [34]. Bybee et al. [35] developed a scale to measure parental guidance in television viewing, which modified has also been applied to other forms of media, such as books and computer games [36]. There are three patterns of parental guidance for television: restrictive, evaluative, and unfocused. Restrictive guidance refers to setting limitations or restrictions on the use of media. For example, parents may set a limit on the amount of time allocated for watching television, and may forbid the watching of particular programs. Evaluative parental guidance means parents will discuss media content with their child; they may also comment on particular content, whether good or bad, and might explain that the content is unrealistic. Unfocused parental guidance refers to a situation where parents only sit near the child while they watch TV and encourage certain watching behavior [35].

### Internet-specific parenting approaches

Parental guidance studied with respect to television is not the same as for Internet use. In the view of Bybee et al. [35], however, several points must be made regarding this application to Internet-specific parenting approaches. The term *informative approaches* will be used instead of *evaluative guidance*; evaluative means a judgment about how well and successfully something is already being done, while *informative* refers to how to do or use something. Parents may not accompany adolescents all the time, and instead may give advice to adolescents before they surf the Internet. In this situation, *informative* is a more suitable descriptor. Moreover, adolescents surf the Internet using a personal computer according to their own wishes, and parents often do not sit next to them while they do this. Therefore, the term *unfocused parental guidance* is replaced by *relational approaches*. A relational approach sees the parent and adolescent use the Internet together to improve bidirectional interaction. This can occur by using the computer to build the parent–child relationship, support each other, and share enjoyment.

### Perceived parental guidance

In this study, the adolescents will perceive parental guidance. According to Van den Bulck and Van den Bergh [36], there are significant discrepancies between the findings drawn from children and parents, as both groups perceive parental intention in a different way. This is because they avoid particular types of guidance and wish to perceive such guidance in an alternative way. It is further suggested that children’s responses to questions are more reliable because parents try to present themselves in a positive light by giving socially desirable answers.

### Family functionality

Family functionality is defined as the promotion of the physical and psychological growth and maturation of all family members [37]. It consists of five components in the Family APGAR Index model: adaptation, partnership, growth, affection, and resolve. This is reported to be useful for Chinese people [38], while the index has been widely used to examine the degree of family functionality in studies related to IA [25, 29, 39]. The degree of family functionality affects general adolescent behavior, as their IA, substance use, and problematic alcohol use are all related to low family functionality [29, 39]. Adolescents with low family functionality are expected to have fewer resources, poorer familial relationships, and less parental support.

### Knowledge gap and aims of the study

To our knowledge, no previous study has examined, as we will, the relationship between parenting approaches and family functionality and adolescent IA. Since Internet use among adolescents is mainly a home-based activity, we hypothesize that parenting approaches and family functionality play a significant role in adolescent IA.

## Methods

A non-experimental approach with a cross-sectional and descriptive comparative design was used to investigate the prevalence of IA and to test the differences in family factors, parenting approaches, and family functionality among the addicted group and the non-addicted group in this study. Written informed consent for participation in the study was obtained from the students and their parent/guardian, where the students were the participants. The research project was approved by the Ethics Committee of the Hong Kong Polytechnic University.

### Participants

Two-stage convenience sampling was used in this study. First, for convenience, two secondary schools were selected from the school list available on the Education Bureau website. In the second stage, students from the schools were recruited if they met the inclusion criteria, which included being ethnically Chinese, aged 12 to 18, and able to read and write Chinese. From this, 2021 students were recruited, and 1248 questionnaire responses that provided usable information were received. The response rate was 62 %. Ultimately, 1163 valid questionnaires remained.

### Questionnaire

Each student completed a questionnaire that included five components assessing demographic information, characteristics of Internet use, an Internet addiction test, parenting approaches to Internet use, and family functionality.

#### Demographic

Respondents were asked to provide their gender, age, education level, guardian/parents’ marital status, total number of children (age ≤ 18) living in the same household, and family income. They were also asked to report how often they had negative feelings towards their parents, and how often their parents had negative feelings towards each other.

#### Characteristics of internet use

Respondents were asked if they had Internet access at home, where they usually used the computer at home, and the average duration of their daily Internet use. Peer influence was measured by asking respondents how often they used the Internet to connect with their friends.

#### Internet addiction test

The 20-item Internet Addiction Test (IAT) developed by Young [23] was adopted to measure the level of Internet use in this study. The test is based on the pathological gambling standards of DSM-IV to develop widely used assessment tools for evaluating IA [40]. In assessing the degree to which respondents’ Internet usage affected their daily routine, productivity, social life, psychological dependence, and time management [41], respondents were asked to rate items on a five-point Likert scale (1 = not at all and 5 = always). The higher the score, the more problematic their Internet use was deemed to be. Young suggested that a score of 20–49 points indicates an average online user who has complete control over their usage; a score of 50–79 reflects frequent problems due to Internet usage; and a score of 80–100 means that the Internet is causing severe problems in the user’s life [23]. In this study, we defined scores of over 50 as being within the addictive group, which corresponds with previous studies [42, 43]. According to Khazaal et al. [43], the split-half reliability and Cronbach’s alpha coefficient of IAT was 0.86 and 0.90 respectively, and it was reported to have high face validity and reliability.

#### Parenting approaches to internet use

The parenting approaches to Internet use were measured in three dimensions: restrictive, informative, and relational; the 13 items were measured using a 4-point scale (1 = never, 2 = seldom, 3 = sometimes, and 4 = often). Items within these three constructs were modified from the Parental Media Guidance scale [36]. The original scale included questions about parental guidance concerning television, computer games, and books; a few adjustments were made to this scale to ensure more appropriate measurements of parenting approaches to Internet use.

#### Family functionality

The 5-item family APGAR score was used to measure the respondents’ level of satisfaction with family functionality. The family APGAR score was designed by Smilkstein [37] to allow respondents to describe feelings of satisfaction with their family’s support in the domains of adaption, partnership, growth, affection, and resolve. The 3-point Likert scale ranged from 0 (hardly ever) to 2 (almost always). The higher the family APGAR scores, the better the family assistance. Respondents with an APGAR score lower than seven were classified as having no family functionality. The reliability of the scale as indicated by Cronbach’s alpha was 0.85, and item-to-total correlations ranged from .50–.65 [36, 44].

### Questionnaire content validity

As the target group of this research included both junior and senior secondary school students, the questionnaire was translated into Chinese to allow for easier reading and understanding. It was translated back into English and compared with the original version to ensure consistency of meaning. All translations were completed by professional translators.

Based on purposeful sampling, two public health nursing researchers and one advanced practice nurse assessed the content validity of the instrument. They were experts in health education and promotion, having extensive research experience with adolescents and health matters. The experts scored the relevancy of each statement using a Likert-type scale. The content validity index (CVI) for each statement was calculated by dividing the number of experts in agreement (who scored 3 and 4 on the Likert scale) by the total number of experts. The statement was accepted if the CVI was greater than 0.79. Ultimately, statements with a CVI lower than 0.79 were modified. The scale-level CVI (S-CVI) was 0.94.

### Statistical analyses

Data analysis was performed using SPSS version 20. Descriptive analysis was used to describe the demographic and Internet-use characteristics of the participants, the prevalence of IA, parenting approaches to Internet use, and family functionality. For inferential statistics, the chi-squared test was applied to evaluate the potential predictors of IA. Factors associated with IA with a *p*-value of less than 0.1 were further considered in the logistic regression analysis. The significance level was set at 0.05.

## Results

### Reliability and validity of the questionnaire

Test-retest reliability was performed by ten respondents that completed the questionnaire twice within a two-week interval. The overall reliability of the whole questionnaire was 0.939. For IAT, its test-retest reliability was 0.95 and internal consistency (Cronbach’s alpha) was 0.89. For parenting approaches to Internet use, its test-retest reliability was 0.89 and internal consistency (Cronbach’s alpha) was 0.81. For family functioning, its test-retest reliability was 0.90 and internal consistency (Cronbach’s alpha) was 0.82.

### Demographic characteristics

Of the 1163 students who had used the Internet, 869 (74.7 %) were normal Internet users, and 294 (25.3 %) were Internet addicted; 460 (39.6 %) students were male, and 703 (60.4 %) were female. Around half of the students were 11–14-year-olds (569; 48.9 %), with the rest (594; 51.1 %) 15–18-year-olds. Approximately half (575; 49.4 %) of the students were in Forms 1–3, while the others (588; 50.6 %) were in Forms 4–6 (Forms 1–7 are equivalent to Grades 7–13 in the United States education system). Around 60 % of students were under both parents’ guidance, and nearly half (535; 46 %) were the only child in the family. Nearly 90 % claimed that their family income was average or above. Just more than two-fifths (487; 41.9 %) stated that they had negative feelings towards their parents, whereas only around a quarter (323; 27.8 %) expressed that their parents had negative feelings towards each other. The detailed statistics can be found in Table 1.

**Table 1:** Comparison of non-internet addicted users and addicted user over the demographic and Internet use characteristics of the participants

		Non-Addicted group (%)	Addicted group (%)	Total (%)	*χ*2
Gender	Female	554 (78.8)	149 (21.2)	703 (60.4)	15.699***
	Male	315 (68.5)	145 (31.5)	460 (39.6)	
Age	11-14	456 (80.1)	113 (19.9)	569 (48.9)	17.326***
	15 -18	413 (69.5)	181 (30.5)	594 (51.1)	
Education^a^	Form 1 - 3	457 (79.5)	118 (20.5)	575 (49.4)	13.629***
	Form 4 - 6	412 (70.1)	176 (29.9)	588 (50.6)	
Guardian	Both parents	542 (76.0)	171 (24.0)	713 (61.3)	1.639
	Others	327 (72.2)	123 (27.3)	450 (38.7)	
Parents’ marital status	Married	812 (76.5)	250 (23.5)	1062 (91.3)	19.577***
	Divorced and others	57 (56.4)	44 (43.6)	101 (8.7)	
Single child family	No	477 (76.0)	151 (24.0)	628 (54.0)	1.102
	Yes	392 (73.3)	143 (26.7)	535 (46.0)	
Family income level	Below average	86 (60.1)	57 (39.9)	143 (12.3)	18. 351***
	Average or above	783 (76.8)	237 (23.2)	1020 (87.7)	
Children having negative emotion to parents	No	572 (84.6)	104 (15.4)	676 (58.1)	83.677***
	Yes	297 (61.0)	190 (39.0)	487 (41.9)	
Parent having negative emotions to parent	No	674 (80.2)	166 (19.8)	840 (72.2)	48.476***
	Yes	195 (60.4)	128 (39.6)	323 (27.8)	
Internet accessibility at home	No	0 (0.0)	0 (0.0)	0 (0.0)	N.A.
	Yes	869 (100)	294 (100)	1163 (100)	
Location of using computer at home	Living room	427 (74.8)	144 (25.2)	571 (49.1)	5.372
	Dining room	37 (64.9)	20 (35.1)	57 (4.9)	
	Bed room	370 (74.9)	124 (25.1)	494 (42.5)	
	Others	35 (85.4)	6 (14.6)	41 (3.5)	
Time spent on internet^b^	<6 h	836 (77.0)	250 (23.0)	1086 (93.4)	44.322***
	≥6 h	33 (42.9)	44 (57.1)	77 (6.6)	
Connect with friends via the Internet	No	147 (82.6)	31 (17.4)	178 (15.3)	6.880**
	Yes	722 (73.3)	263 (26.7)	985 (84.7)	
Overall		869 (100)	294 (100)	1163 (100)	

**p <0.01; ***p <0.001; ^a^Form 1–7 is equivalent to year 7–13 in US education system; ^b^It will not be further considered in regression analysis for preventing the problem of over controlling for factors

The results of the chi-square analysis show that there are significant differences between the addicted and non-addicted groups when considering the factors of gender, age, education, parents’ marital status, family income, children with negative feelings towards their parents, and parents with negative feelings towards each other (*p* < 0.001). More statistical information can be found in Table 1.

### Internet usage characteristics

All students reported using the Internet at home, and most of them used a computer in their living room (571; 49.1 %) or bedroom (494; 42.5 %). A significantly high proportion of students who spent more than six hours per day on the Internet belonged to the addicted group (*p* < 0.001). Moreover, students who connected with friends via the Internet also had a higher percentage of IA (*p* < 0.01), as shown in Table 1.

### Parenting approaches and family functioning

Table 2 shows the comparison of parenting approaches and family functionality between the addicted and non-addicted groups. The results show that all three approaches were popular: 755 restrictive (64.9 %), 761 informative (65.4 %), and 638 rational (54.9 %). However, a significantly higher proportion of students in families that used the restrictive approach belonged to the addicted group (*p* < 0.05). In terms of family functionality, it was found that students in the severely or moderately dysfunctional groups were more likely to belong to the addicted group (*p* < 0.001).

**Table 2:** Comparison of non-internet addicted users and addicted user over the parenting approaches and family functioning characteristics of the participants

		Non-addicted group (%)	Addicted group (%)	Total (%)	*χ*2
Restrictive approach	No	323 (79.2)	85 (20.8)	408 (35.1)	6.577*
	Yes	546 (72.3)	209 (27.7)	755 (64.9)	
Informative approach	No	302 (75.1)	100 (24.9)	402 (34.6)	0.053
	Yes	567 (74.5)	194 (25.5)	761 (65.4)	
Relational approach	No	392 (74.7)	133 (25.3)	525 (45.1)	0.001
	Yes	477 (74.8)	161 (25.2)	638 (54.9)	
Family functioning	Severely dysfunctional	90 (61.6)	56 (38.4)	146 (12.6)	30.651***
	Moderately dysfunctional	371 (71.3)	149 (28.7)	520 (44.7)	
	Highly functional	408 (82.1)	89 (17.9)	497 (42.7)	

*p <0.05; ***p <0.001

### Logistic regression analysis for IA

Table 3 shows the results of analyses of the association between family factors and IA by logistic regression. The analyses were adjusted for covariates, including gender, age, parents’ marital status, and family income level, which were significant predictors of IA. Regarding other predictors, adolescents that had negative feelings towards their parents were found to be almost three times more likely to have IA (OR: 2.903; *p* < 0.001). Parents having negative feelings towards each other also implied a significantly higher probability of IA (OR: 1.498; *p* < 0.05). The results also showed that adolescents in a family that used the restrictive parenting approach were almost 1.9 times more likely to have IA (OR: 1.857; *p* < 0.001). A severely dysfunction family was also one of the predictors of adolescents being predisposed to IA (OR: 1.935; *p* < 0.01) when compared to highly functional families.

**Table 3:** Logistic regression analysis for the family predictors for IA

	Variables	OR	95 % CI
Gender	female	1	
	male	1.839	1.368-2.473***
Age	11-14		
	15 -18	2.005	1.476-2.723***
Parents’ marital status	Married	1	
	Divorced or others	2.536	1.582-4.063***
Family income level	Average or above	1	
	Below average	1.872	1.240-2.826**
Connect with friends via the Internet	No	1	
	Yes	1.978	1.254-3.118**
Children having negative emotion to parents	No	1	
	Yes	2.903	2.111-3992***
Parent having negative emotion to parent	No	1	
	Yes	1.498	1.083-2.073*
Restrictive parenting approaches	No	1	
	Yes	1.857	1.341-2.572***
Family functioning	High functional	1	
	Moderately dysfunctional	1.278	0.922-1.771
	Severely dysfunctional	1.935	1.227-3.054**

*p <0.05; **p <0.01; ***p <0.001

## Discussion

### Prevalence of internet addiction

The prevalence of IA among Hong Kong adolescents in this study was 25.3 %, a finding that demonstrates that this addiction is increasing; a previous study found a prevalence of 19.1 % in 2008 [12]. This increase, within only four years, is an alarming signal and should not be neglected. In contrast, statistics in China indicate that the prevalence of IA is 10.8 % among adolescents aged 13–18 years of age [9], much lower than in Hong Kong. This is probably due to the poor Internet coverage in rural China and because of poor Internet connection speeds.

### Gender

This study found a relationship between being male and IA. Several previous studies reported being male as one of the IA risk factors [6, 7, 45], relating the widespread popularity of online gaming, gambling, and pornographic material to the high prevalence of IA among males [6, 46]. However, research also suggests that gender is not related to IA [11, 15, 47]. In a recent study, Shek and Yu [48] claimed, after reviewing the previous research reports, that there is no agreement among researchers on gender differences in IA. Hence, it is necessary to investigate further to clarify any gender effects. It is hypothesized that there was no gender difference in IA, but different genders might more easily become addicted to different types of electronic device.

### Age

Late adolescence (youths aged 15–18) in this study indicated a significantly higher incidence of IA compared to early adolescence (youths aged 11–14). Ko et al. [46] mentioned that the factor of age was positively associated with the level of IA in a sample of junior high school male students. Although Lam et al. [9] and Lin et al. [15] also studied the relationship between age and IA among adolescents, their findings did not demonstrate that age was significantly associated with IA level.

### Family income

Low family income was found to be a predictor of IA. Adolescents from families with a low household income received less resources and support for their needs, and surfing the Internet is certainly an inexpensive alternative activity. Lower-income families generally have lower educational achievements, meaning parents in such families may not know of the adverse effects of IA or the strategies available to prevent it from occurring, such as good communication and relationship building.

### Parents’ marital status

Being an adolescent with divorced parents was a strong predictor of IA. However, it is suggested that there is no significant difference between having married or divorced parents [29]. In a divorced family, a single parent needs to support the entire family, which means there is limited time to build a relationship with the children. In addition, adolescents in divorced families may resort to accessing the Internet to relieve the psychological insecurities that develop in a single-parent family environment [49].

### Connecting with friends

Using the Internet to connect with friends was a significant predictor of IA in this study. A previous study also supported the view that seeking online interaction and friendship can lead to IA [50]. The Internet is a convenient tool for adolescents to pursue peer relationships. Thus, many friendships are formed online, as the Internet does not place many restrictions upon them when they interact. However, the overuse of the Internet for social interaction may result in IA [51].

### Family conflict and severe family dysfunction

This study shows that high levels of family conflict and severe family dysfunction strongly predict IA, results consistent with previous findings [25, 29]. According to the developmental model of adolescent problem behavior [52], families with high levels of conflict were likely to have low levels of family involvement, resulting in inadequate parental monitoring. This in turn is a predisposing factor in problematic behavior. Furthermore, the social control theory states that when adolescents have greater attachment to their parents and positive family interactions, they feel obligated to act in non-deviant ways to please their parents [53]. In contrast, adolescents in families with high levels of conflict and poor family bonding would refuse parental supervision or monitoring.

Severe family dysfunction implies inadequate familial resources, fewer instances of shared parental decision making and nurturing responsibilities, a lack of mutual support and guidance, relationships deficient in love or care, and insufficient devotion of time to other family members. In Hong Kong, most parents need to work day and night, resulting in a lack of awareness of the needs of their adolescent children. Davis [54] mentioned that when parents pay insufficient attention and there is a lack of parental support, adolescents are more likely to be psychologically unstable. In an environment with fierce social competition, parents mainly focus on the academic achievements of their adolescent children. Commonly subjected to great pressure and high expectations, adolescents may grow up without parental warmth. To compensate for this psychological deficiency, they maintain their self-fulfillment, build relationships, and gain a temporary sense of affection, inclusion, and belonging through the virtual world of the Internet [55]. Addiction to these positive feelings contributes to the risk of IA.

### Parenting approaches to adolescents’ internet use

The restrictive parenting approach was found to be significantly associated with adolescent IA; the greater the number of rules, and the stricter the enforcement of rules concerning Internet use, the more likely it is that adolescents will become addictive users. It has been identified that IA shares similar characteristics with other problematic addictive behaviors, such as alcoholism, drug abuse, and pathological gambling [56]. It is notable that our findings contrasted with the results of studies focusing on alcohol-specific restrictive parenting approaches, which showed that parental rules about alcohol consumption were effective in preventing adolescents from developing early drinking behavior [57–59]. Van den Eijnden et al. [60] suggested that parental control over the time spent using the Internet might promote the development of adolescent IA, which is in line with our findings. As adolescents grow up, they want more autonomy and independence. Therefore, the use of the most unpopular parenting approach, the restrictive approach, may be counterproductive, and adolescents may act in a deviant way as a coping mechanism for dealing with stressful daily life events [61].

Informative and relational parenting approaches were not significantly related to IA in this study, with the results similar to those of Van den Bulck and Van den Bergh [36]. These approaches focus on parent–child involvement and communication. However, neither approach guarantees the quality of communication between parents and adolescents. The authors suggested that the quantity of time spent together and frequency of communication regarding Internet use were not associated with adolescent IA, but that quality of communication was. Only high-quality communication was a promising tool for preventing adolescents from becoming addicted to the Internet [7, 60]. Nevertheless, the findings on parenting approaches are consistent with Floros and Siomons’ study, which suggested that parents caring for and protecting their children, while respecting their autonomy, will reduce adolescents’ motivation to become involved with social networking participation and IA [62].

### Limitations

The results of this study should be considered in light of its limitations. First, the cross-sectional research design could not confirm whether causal relationships between IA and possible influential factors existed. Longitudinal studies are needed to examine adequately the direction of the effects. Second, the use of convenience sampling limits generalizability and induces bias. Third, the influential factors are perceived solely based on adolescents’ self-reported data, and there is a lack of information from the parents’ perspective. Fourth, the pressure to provide socially desirable responses concerning Internet use may make adolescents unwilling to reveal their true usage, even in anonymous questionnaires. Fifth, the study could not include all possible factors. For example, considering the motives for Internet use could be meaningful in future studies, as Floros and Siomons showed that adolescents’ motives for participating in social networking are significantly related to parenting style and IA [62]. Hence, further studies should attempt to determine additional factors.

## Conclusions

Adolescents are at a stage of life during which they experience significant changes biologically, psychologically, and socially. Those who have trouble controlling their online activities are particularly vulnerable to IA. However, parents can play a protective role in their response to adolescent behavior, and the importance of family functionality and parenting approaches should not be underestimated. Family-based prevention of IA should be implemented to deal with this globally problematic issue. Nevertheless, as this study only included students from two schools in Hong Kong, further studies are required to improve the generalizability of the findings.

Based on the results of this study and previous reports [7, 63], it is clear that positive family relationships and interaction play an important role in preventing IA. It is suggested that family-based interventions should include improving parents’ communication proficiency and fostering the skills required to achieve healthy family interactions and strengthen family functionality, rather than directly restricting Internet use.

## Abbreviations

CVI, content validity index, IA, internet addiction, IAD, internet addiction disorder, IAT, internet addiction test
